# Interpersonal trust and objectively measured physical activity in adolescents: the mediating role of family capital

**DOI:** 10.3389/fpubh.2026.1796300

**Published:** 2026-04-24

**Authors:** Lan Luo, Fawei Huang, Syeda Rabia Tahir, Hanchen Shao, Baixia Li

**Affiliations:** 1 Key Laboratory of Intelligent Sports with Medicine and Active Health for All Ages, School of Physical Education and Health, Sichuan Technology and Business University, Meishan, Sichuan, China; 2 Faculty of Education and Humanities, UNITAR International University, Petaling Jaya, Malaysia; 3 City University Malaysia, Petaling Jaya, Malaysia; 4 East China University of Science and Technology, Shanghai, China

**Keywords:** adolescents, family capital, interpersonal trust, objectively measured physical activity, social capital, structural equation modeling

## Abstract

**Purpose:**

Grounded in social capital theory and the family ecological systems framework, this study examined whether interpersonal trust was statistically associated with objectively measured physical activity among adolescents, and whether perceived family capital accounted for part of this association. Identifying psychosocial correlates of adolescent physical activity is important for informing theory-based public health strategies.

**Methods:**

A cross-sectional study was conducted with 358 secondary school students in Sichuan Province, China. Participants wore ActiGraph wGT3X-BT accelerometers for seven consecutive days. Accelerometer data were processed using a standardized and transparent workflow to derive moderate-to-vigorous physical activity (MVPA). After quality control procedures, 326 participants were included in the analysis. Interpersonal trust and family capital (economic, social, and cultural dimensions) were assessed using validated questionnaires. Structural equation modeling was used to estimate direct and indirect statistical associations.

**Results:**

Interpersonal trust was positively associated with MVPA (β = 0.20, *p* < 0.001), and perceived family capital was also positively associated with MVPA (β = 0.39, *p* < 0.001). Perceived family capital demonstrated a significant indirect statistical association in the relationship between interpersonal trust and MVPA [indirect β = 0.19, 95% CI (0.12, 0.26)], accounting for 48.7% of the total standardized association.

**Conclusions:**

These findings are consistent with a relational pattern linking psychosocial factors and objectively measured physical activity within the family ecological context. Given the cross-sectional design, the observed associations should not be interpreted as evidence of temporal or causal relationships.

## 1 Introduction

Insufficient physical activity is widely acknowledged as a significant global public-health challenge. The World Health Organization has noted that the majority of adolescents across the globe do not meet the recommended daily levels of moderate-to-vigorous physical activity (MVPA) (1). Physical activity levels during adolescence are not only closely correlated with current body-weight status, metabolic health, and mental health but may also impact chronic-disease risk and health-behavioral trajectories in adulthood. Therefore, identifying the key social and psychological factors influencing adolescent physical activity is a crucial research topic in public health.

According to the Social Ecological Model, health behaviors are embedded within a multi-layered social-environment structure encompassing the family, peer group, school, and community (2). In recent years, social capital has gained increasing attention as a key theoretical framework for explaining health disparities and health behaviors (3). Systematic reviews indicate statistical associations between social capital—particularly at the family and community levels—and adolescent health-risk behaviors and physical activity levels (4, 5). However, two notable gaps remain. First, most existing studies rely on self-reported questionnaires to assess physical activity, which may introduce recall bias and social desirability bias (6). Second, few studies have simultaneously examined specific psychological dimensions of social capital—such as interpersonal trust—in relation to objectively measured physical activity among adolescents (7). This gap limits our understanding of how distinct psychosocial components of social capital relate to objectively assessed health behaviors.

Interpersonal trust is typically defined as an individual's expectation of others' reliability and benevolence, serving as a crucial psychological dimension of social capital. Research in developmental neuroscience and developmental psychology indicates that adolescence represents a critical stage for the gradual stabilization of social cognition and trust structures (8). Trust levels are significantly associated with an individual's social participation, cooperative behaviors, and frequency of group interactions (9).

Physical activity, particularly team sports and extracurricular athletic participation, typically involves a high degree of social interaction (10). Therefore, it is theoretically plausible that levels of interpersonal trust may exhibit a statistical association with adolescents' participation in physical activity (11). However, current empirical research on the relationship between interpersonal trust and physical activity remains limited. Most studies rely on self-reported physical activity measures with insufficient evidence supported by objectively measured physical activity data. These observations raise an important question regarding the potential mechanisms through which interpersonal trust may relate to adolescent physical activity.

Within the framework of social-capital theory, family capital is typically regarded as a crucial structural resource influencing adolescent development, encompassing dimensions such as economic capital, social capital, and cultural capital (4, 11). Family resources may be associated with physical activity levels through multiple pathways, including providing sports facilities, covering the costs of athletic training, building supportive social networks, and shaping values regarding healthy behaviors (12).

In physical-activity research, the statistical mediating role of psychosocial variables has gradually gained attention (13). For instance, factors such as family support, self-efficacy, and peer support may form associative structures between social environments and physical activity (2). However, empirical testing of the theoretical pathway linking interpersonal trust, family capital, and physical activity remains limited, particularly in the context of objectively measured physical activity.

It is noteworthy that while family capital is often theoretically regarded as a precursor to adolescents' psychological and behavioral development, dynamic or reciprocal processes may also exist between psychological traits and family interaction structures during adolescence. Therefore, when testing theoretically consistent models within a cross-sectional design, caution should be exercised in interpreting the directionality of relationships between variables.

### 1.1 Theoretical model and research objectives

Drawing on social capital theory and developmental perspectives, we specify a theory-informed structural model to examine the statistical associations among interpersonal trust, perceived family capital, and objectively measured moderate-to-vigorous physical activity (MVPA).

It is important to clarify the theoretical positioning of this model specification. Within classical social capital theory, family capital—particularly its economic and structural dimensions—is commonly conceptualized as an antecedent condition shaping adolescents' psychosocial development, including the formation of interpersonal trust. From this perspective, family capital may precede and statistically relate to interpersonal trust rather than result from it.

However, during adolescence, psychosocial dispositions and family resource structures may operate in dynamic and potentially reciprocal ways. In the present study, family capital was operationalized as perceived family capital, reflecting adolescents' subjective evaluations of family economic, social, and cultural resources. Such perceptions may be influenced not only by objective household conditions but also by relational experiences and interpersonal orientations. Adolescents with higher interpersonal trust may interpret family interactions, support, and available resources more positively, which may be reflected in higher reported perceived family capital.

**Figure 1:**
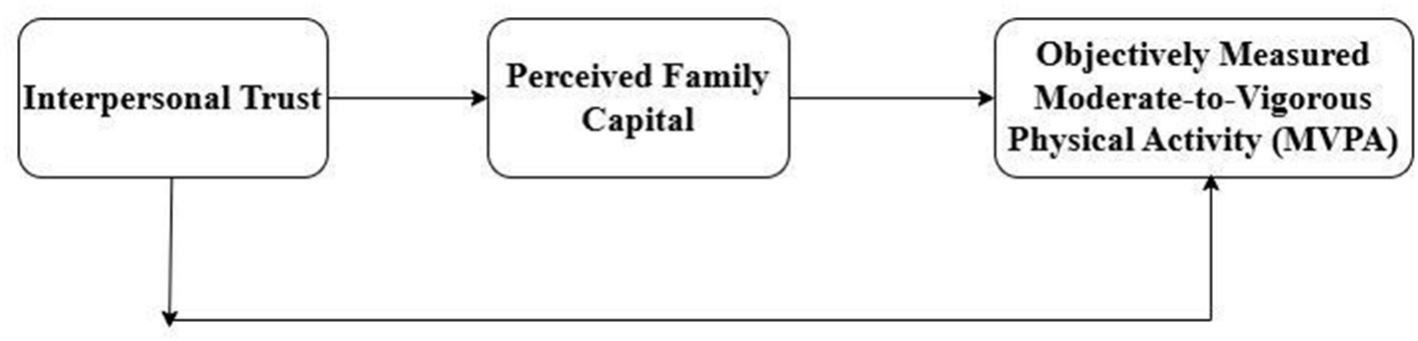
Theory-informed structural model of statistical associations.

Accordingly, the structural model specified in this study does not assume that objective family resources are causally generated by interpersonal trust. Rather, it represents a theory-informed statistical structure in which interpersonal trust and perceived family capital are conceptualized as interrelated components within a broader social-ecological system. Given the cross-sectional design, the direction of the specified paths reflects theoretical ordering rather than established temporal sequence. Alternative configurations—such as perceived family capital statistically predicting interpersonal trust, or reciprocal statistical associations between the constructs—are theoretically plausible and warrant examination in future longitudinal or cross-lagged research designs. Therefore, the present statistical mediation model should be interpreted as representing a statistical pattern consistent with the hypothesized pathway, rather than evidence of a unidirectional causal mechanism. The conceptual structure of the proposed statistical associations is illustrated in Figure 1.

### 1.2 This study aims to:

(1) Examine the statistical association between interpersonal trust and objectively measured moderate-to-vigorous physical activity (MVPA).
(2) Examine whether perceived family capital demonstrates statistical mediation within this association.
(3) Provide theory-informed empirical evidence on the statistical associations between psychosocial factors and objectively measured MVPA.

Given the cross-sectional design of this study, the proposed model reflects statistical associations among variables rather than temporal ordering or causal processes.

## 2 Methods

### 2.1 Study design and setting

This study employed a cross-sectional design to examine the statistical associations among adolescents' interpersonal trust, perceived family capital, and objectively measured physical activity. The research was conducted in two secondary schools in Sichuan Province, China.

Research participants were recruited through convenience sampling. The inclusion of two schools was primarily driven by operational feasibility, including device availability and staffing capacity required for standardized accelerometer deployment and data management. While the schools were not randomly selected, efforts were made to ensure consistent implementation procedures. Accordingly, the findings should be interpreted within the context of school-based observational research and warrant replication in broader and more diverse settings.

Following school approval, the research team conducted recruitment in accordance with ethical review procedures. After obtaining written informed consent from parents or legal guardians and assent from students, a total of 401 adolescents were enrolled in the study. All participants wore accelerometers for 7 days during the regular academic semester and completed questionnaires on interpersonal trust and family capital. This study did not involve any interventions or experimental procedures; all variables were observed and measured in natural settings.

### 2.2 Participants and sampling

The study subjects were students aged 12–17 enrolled in two secondary schools in Sichuan Province. Students voluntarily enrolled through class announcements and parent-school communication channels. Inclusion criteria were: (1) ability to participate in normal daily physical activities; (2) agreement to wear an accelerometer continuously for 7 days; (3) completion of relevant questionnaires. Exclusion criteria included health conditions affecting normal physical activity or failure to meet accelerometer data quality standards.

Following completion of the ethics review and informed consent procedures, a total of 401 students were initially enrolled in the study. All participants were provided with accelerometers and completed questionnaires. Among them, 358 participants successfully returned the devices and yielded downloadable accelerometer data. Based on predefined data quality criteria (detailed in Section 2.4), 32 participants were excluded from the final analysis sample for failing to meet valid wear time. Specific reasons included device malfunction (*n* = 8), failure to meet the minimum number of valid monitoring days (*n* = 18), and excessive non-wear periods (*n* = 6). Ultimately, data from 326 participants met the predefined standards and were included in the statistical analysis.

To assess whether sample exclusion might exert a systematic influence on sample structure, this study compared differences in demographic and psychosocial variables between samples included in the analysis and those excluded. The relevant results are presented in Table 1.

**Table 1:** Comparison between included and excluded participants.

Variable	Included(*n* = 326)	Excluded(*n* = 32)	Test statistic	*p* value
Age (coded), mean ± SD	3.11 ± 1.34	3.06 ± 1.52	*t* = 0.16	0.873
Male sex, *n* (%)	193 (59.2%)	18 (56.2%)	χ^2^ = 0.02	0.892
Interpersonal trust, mean ± SD	3.42 ± 0.68	3.14 ± 0.63	*t* = 2.45	0.015
Family capital, mean ± SD	3.67 ± 0.71	3.11 ± 0.64	*t* = 3.12	0.002

The demographic characteristics and key study variables of the final analytic sample (*N* = 326) are presented in Table 2. The sample included 193 male participants (59.2%), consistent with the class composition of the participating schools during the recruitment period. Gender was included as a covariate in subsequent structural models to account for potential sex-related differences.

**Table 2:** Descriptive statistics of key study variables (*N* = 326).

Variable	Mean ±SD	Range
Interpersonal trust	3.42 ± 0.68	1.45–4.95
Family capital	3.67 ± 0.71	1.80–5.00
MVPA (min/day)	47.3 ± 22.1	8.5–128.4
Steps/day	8,247 ± 2,834	2,145–16,892
Sedentary time (h/day)	8.9 ± 1.6	4.2–13.1

Given that this study employed convenience sampling and drew its sample from two schools, the findings primarily reflect statistical associations between variables within a specific educational context. Caution is advised when extrapolating these results to other regions or different sociocultural settings.

### 2.3 Wearable technology

**Figure 2:**
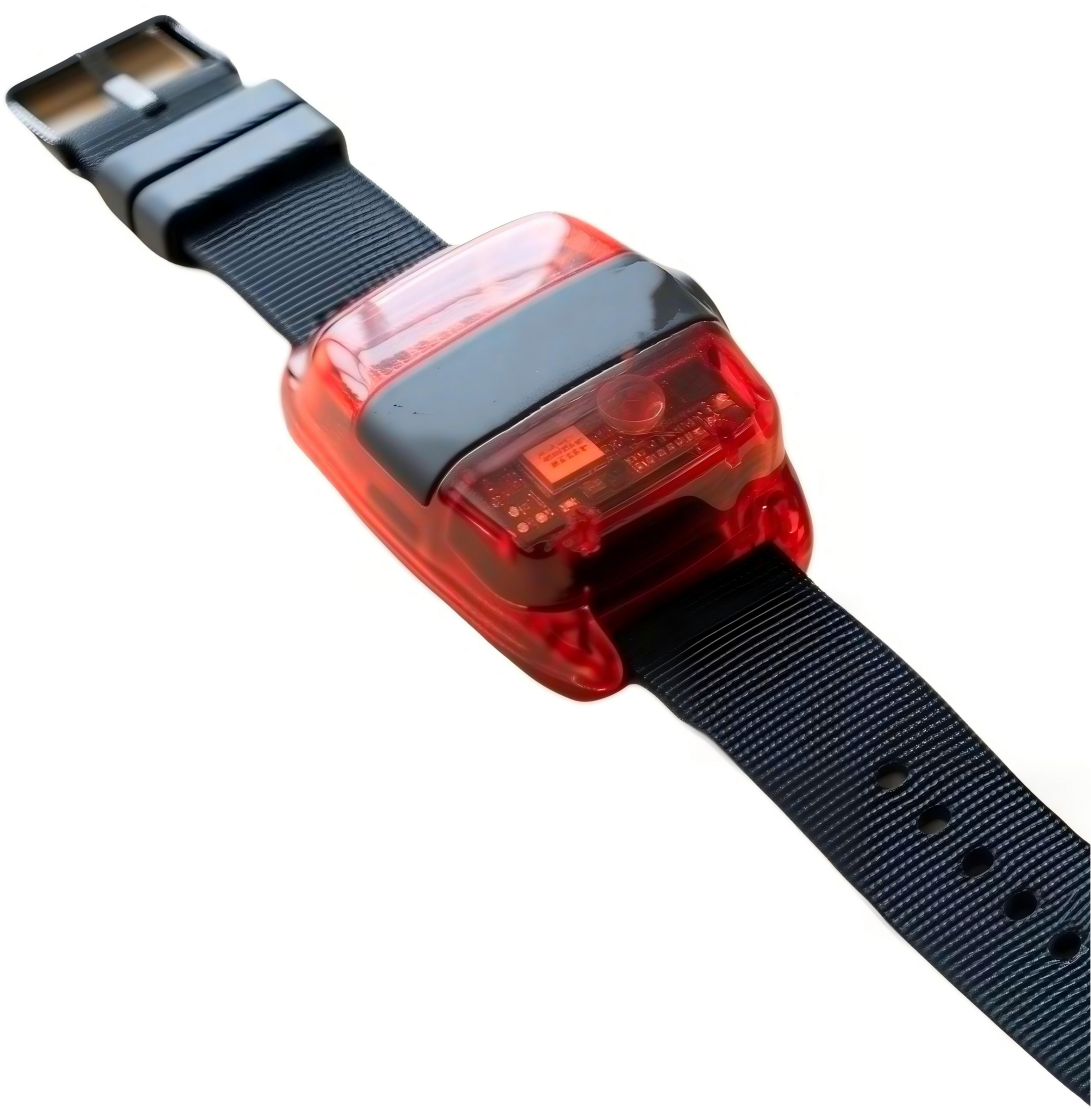
The ActiGraph wGT3X-BT accelerometer used in the study.

The ActiGraph wGT3X-BT accelerometer was initialized at a sampling frequency of 30 Hz and deployed for seven consecutive days of monitoring. Hip placement was selected because the count-based intensity thresholds applied in this study (Evenson cut-points) were validated primarily for hip-worn ActiGraph devices in pediatric populations. Participants were instructed to wear the device during waking hours (6:00–23:00) throughout the monitoring period to balance measurement validity and feasibility in the school setting. This wear protocol may not capture early-morning or late-evening activity and may result in underestimation if the device was removed for water-based activities or comfort. Raw acceleration data were downloaded using ActiLife software (version 6.13.4) and processed into analysis-ready files. The device is shown in Figure 2.

### 2.4 Wearable deployment procedures

Following device configuration, standardized deployment procedures were implemented as described below. To enhance compliance, homeroom teachers provided reminders during the initial wearing period and issued a unified reminder midway through the monitoring cycle. No financial incentives were offered in this study. Devices were uniformly collected in class 1 week later.

Among the initial sample of 401 participants, 358 returned the devices (89.3%). Of these, 326 met the criteria for valid wear time and were included in the analysis. Exclusions primarily resulted from insufficient wear duration or failure to meet the valid monitoring days, with a small number attributed to device malfunctions. Individual students reported minor discomfort from the waistband or instances of forgetting to reattach it.

The device does not enable location tracking and only records motion acceleration signals. After data download, it undergoes de-identification processing and is stored on an encrypted server. The school does not access individual data.

We did not formally assess day-of-wear reactivity (e.g., by comparing MVPA on day 1 vs. subsequent days or conducting sensitivity analyses excluding the first monitoring day). As a result, potential short-term behavioral changes related to being monitored cannot be ruled out and should be examined in future studies using explicit day-specific analyses.

Three trained research assistants handled device distribution and collection. Introducing the devices and explaining how to wear them usually took about two class periods. Collecting the devices at the end of the week required roughly one class period. The devices were worn using adjustable elastic belts. No formal fitting procedure was needed, although a few students mentioned mild discomfort from the waistband on the first day. Teachers helped maintain compliance by reminding students during the first days of monitoring and once again midway through the week. When students forgot to reattach the device after removing it, the issue was usually resolved with a short reminder in class.

### 2.5 Accelerometer data collection and processing

**Figure 3:**
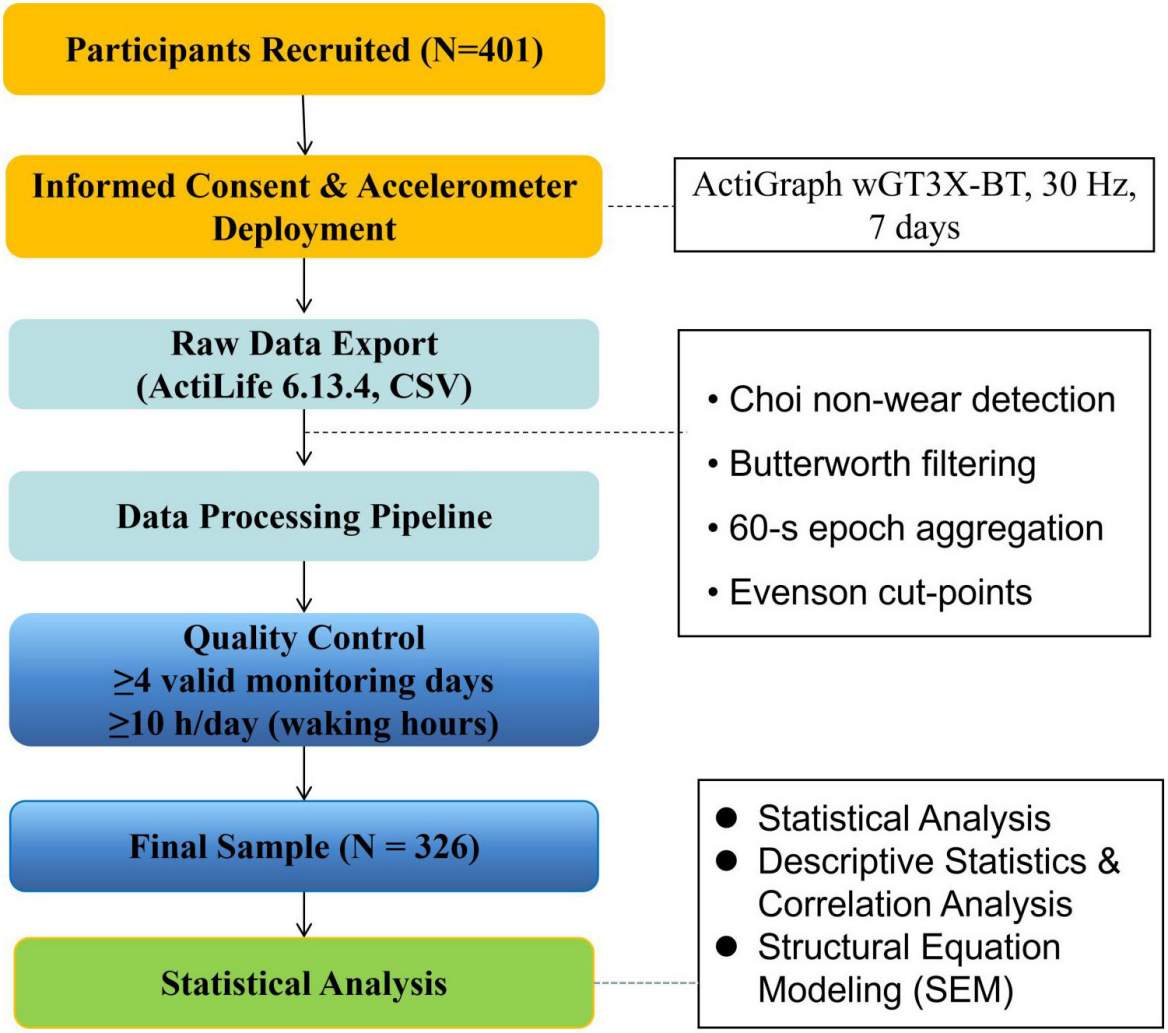
Study design and data processing workflow.

To ensure standardized and reproducible estimation of activity intensity metrics, accelerometer data were processed according to a pre-defined workflow (Figure 3). Raw accelerometer data were downloaded using ActiLife software (version 6.13.4). Activity counts were generated within ActiLife and summarized into 60-second epochs for activity processing. Non-wear time was identified on the epoch-level count data using the algorithm proposed by Choi et al. (14), defined as ≥90 consecutive min of zero counts with allowance for short interruptions. Physical activity intensity was classified using the Evenson cut-points validated for children and adolescents, with thresholds defined as sedentary (0–99 counts/min), light (100–2,295 counts/min), moderate (2,296–4,011 counts/min), and vigorous (≥4,012 counts/min) (15).

Moderate-to-vigorous physical activity (MVPA) was calculated as the combined duration of moderate and vigorous activity. A valid monitoring day was defined as at least 10 h of wear time during waking hours (6:00–23:00), and participants were required to provide at least four valid days, including one weekend day. These criteria are consistent with established recommendations in pediatric accelerometry research and represent a balance between data quality and participant retention. Core accelerometer processing procedures are summarized in Supplementary Appendix S1.

### 2.6 Psychosocial measures

#### 2.6.1 Interpersonal trust scale

The Chinese version of Rotter's Interpersonal Trust Scale (ITS) was employed (16), following prior validation studies in Chinese adolescent samples. The scale covers both general and specific trust dimensions. Items were rated on a 5-point Likert scale (1 = strongly disagree to 5 = strongly agree). Example items: “Most people can be trusted,” “People are generally honest.” The Cronbach's α coefficient in this study was 0.937, indicating excellent internal consistency. To ensure suitability for adolescent respondents, items referring to complex institutional contexts or adult social roles were excluded.

#### 2.6.2 Family capital questionnaire

The Family Capital Scale was assessed using the instrument reported by Sui and Huang (17), which was constructed based on the Social Capital Factor Scale and the Measurement of Cultural Capital in China Scale. The instrument comprises three dimensions—family economic, social, and cultural capital—with items rated on a 5-point Likert scale (see Table 3). Internal consistency in the present sample was good (Cronbach's α = 0.855). In this study, family capital reflects adolescents' perceived family resources rather than objectively measured socioeconomic indicators (e.g., household income or parental education). Although the economic dimension captures perceived material resources, it does not represent objective socioeconomic status. This subjective operationalization aligns with a social-ecological framework but may involve shared perceptual variance with interpersonal trust; therefore, structural associations should be interpreted cautiously.

**Table 3:** Structure and measurement of the family capital questionnaire.

Dimension	Definition	No. of items	Sample item^*^	Scale source	Response format
Family economic capital	Reflects adolescents' perceived material resources and economic conditions of the family	5	“My family's economic condition is better than that of most families with similar backgrounds.”	Family capital scale (18)	5-point Likert scale (1 = strongly disagree, 5 = strongly agree)
Family social capital	Captures adolescents' perceived family social networks, relational resources, and external support	5	“My family has extensive social connections that can provide help when needed.”	Family capital scale (18)	5-point Likert scale
Family cultural capital	Represents adolescents' perceived educational resources, cultural atmosphere, and learning support within the family	5	“My family places great importance on education and cultural activities.”	Family capital scale (18)	5-point Likert scale
Overall perceived family capital	Comprehensive latent construct representing adolescents' perceived family resources	15	—	Adapted from Sui and Huang, 2025 (18)	Cronbach's α = 0.855

^*^Sample items are illustrative examples of each dimension; the full questionnaire includes multiple items.

### 2.7 Statistical analysis

Data were analyzed using SPSS 26.0 and Mplus 8.3. All tests were two-tailed, and statistical significance was set at *p* < 0.05. Given the cross-sectional design, relationships among variables were interpreted as statistical associations without implying temporal ordering or causal inference.

Descriptive statistics, including means and standard deviations, were calculated for demographic characteristics, psychosocial variables, and objectively measured physical activity indicators. To evaluate potential selection bias resulting from sample screening, independent samples *t*-tests and χ^2^ tests were conducted to compare demographic and psychosocial characteristics between the final analytic sample and excluded participants. Pearson correlation analyses were then performed to examine bivariate statistical associations among the primary study variables, providing the basis for subsequent structural modeling.

Prior to estimating the structural model, confirmatory factor analysis (CFA) was conducted to assess the measurement properties of the interpersonal trust and family capital constructs. Model fit was evaluated using multiple fit indices, including the χ^2^/df ratio (< 3.0), Comparative Fit Index (CFI >0.90), Tucker–Lewis Index (TLI >0.90), Root Mean Square Error of Approximation (RMSEA < 0.08), and Standardized Root Mean Square Residual (SRMR < 0.08). Convergent validity was examined through standardized factor loadings, composite reliability (CR), and average variance extracted (AVE) (18). Discriminant validity was assessed by comparing the square roots of AVE values with inter-construct correlations. Given that psychosocial variables were measured via self-report, potential common method bias was evaluated using Harman's single-factor test and by comparing a single-factor CFA model with the hypothesized multi-factor measurement model (19).

Structural equation modeling (SEM) was subsequently employed to examine statistical associations among interpersonal trust, family capital, and objectively measured moderate-to-vigorous physical activity (MVPA). The model estimated associations between interpersonal trust and family capital, between family capital and MVPA, and the direct association between interpersonal trust and MVPA. Gender, age, and grade level were included as covariates to reduce potential confounding. Indirect effects were estimated using bias-corrected bootstrap procedures with 5,000 resamples, and 95% confidence intervals were reported. Consistent with the cross-sectional design, indirect effects were interpreted as statistical indirect associations rather than evidence of causal mediation.

To assess the robustness of the findings, sensitivity analyses were conducted by examining weekday and weekend MVPA separately and by additionally adjusting for sedentary time within the structural model. Accelerometer data completeness was determined during pre-processing based on pre-defined wear-time validity criteria. Questionnaire missingness was minimal (3%) and was addressed using full information maximum likelihood (FIML) estimation to reduce potential bias in parameter estimates (20).

Objective socioeconomic indicators, such as parental education or household income, were not collected. Accordingly, residual confounding related to socioeconomic position cannot be excluded, and perceived family capital may partly capture broader structural socioeconomic influences. Future research should incorporate objective socioeconomic indicators (e.g., parental education, household income) to disentangle perceived family capital from structural socioeconomic position. To examine whether the results were influenced by potential school-level differences, an additional sensitivity structural equation model adjusting for school (two-school indicator) was conducted.

### 2.8 Ethics statement

This study was reviewed and approved by the Ethics Committee of Sichuan Technology and Business University (Approval No. STBU-EC-2025-008). Written informed consent was obtained from the parents or legal guardians of all participants. Adolescents provided assent before taking part in the study.

## 3 Results

### 3.1 Participant flow and data completeness

A total of 401 students were initially enrolled in the study. Of these, 358 participants (89.3%) returned the accelerometers with downloadable data. Based on the pre-specified wear-time validity criteria, 32 participants were excluded due to insufficient monitoring data (device malfunction, *n* = 8; insufficient valid days, *n* = 18; excessive non-wear periods, *n* = 6), resulting in a final analytic sample of 326 adolescents.

To evaluate potential selection bias, demographic and psychosocial characteristics were compared between included and excluded participants (Table 1). No significant differences were observed in age (*p* = 0.873) or gender distribution (*p* = 0.892). However, excluded participants reported significantly lower levels of interpersonal trust (*p* = 0.015) and perceived family capital (*p* = 0.002) compared to those retained in the analytic sample.

These findings suggest that adolescents with lower psychosocial resource levels may have been less likely to meet accelerometer wear-time criteria. Accordingly, the final analytic sample may modestly overrepresent students reporting higher levels of interpersonal trust and perceived family capital, which should be considered when interpreting the magnitude and generalizability of the reported associations.

Participants in the analytic sample provided an average of 6.2 ± 0.9 valid monitoring days, with 14.3 ± 1.8 h/day of wear time under the waking-hours protocol. Detailed accelerometer quality metrics are presented in Supplementary Table S1.

### 3.2 Descriptive statistics and correlations

Descriptive statistics for the final sample (*N* = 326) are presented in Table 2. The sample included 193 males (59.2%) and 133 females (40.8%), with a mean age of 14.10 ± 1.36 years (range: 12–17 years). Participants accumulated an average of 47.3 ± 22.1 min per day of moderate-to-vigorous physical activity (MVPA). Mean interpersonal trust was 3.42 ± 0.68, and mean perceived family capital was 3.67 ± 0.71.

Pearson correlation analyses showed statistically significant positive associations among the primary variables. Interpersonal trust was positively correlated with MVPA (*r* = 0.34, *p* < 0.001) and with family capital (*r* = 0.49, *p* < 0.001).

Family capital was also positively correlated with MVPA (*r* = 0.41, *p* < 0.001). These associations provided preliminary support for the hypothesized structural relationships. Independent-samples tests indicated no statistically significant gender differences in MVPA (males: 48.1 ± 23.2 vs. females: 45.8 ± 20.1 min/day, *p* = 0.36), interpersonal trust (*p* = 0.45), or family capital (*p* = 0.51).

### 3.3 Measurement model (confirmatory factor analysis)

Prior to estimating the structural model, confirmatory factor analysis (CFA) was conducted to evaluate the measurement properties of interpersonal trust and perceived family capital. The measurement model demonstrated acceptable fit to the data, supporting the adequacy of the latent construct specification.

All standardized factor loadings were statistically significant (*p* < 0.001). Composite reliability (CR) values exceeded 0.70 for all constructs, and average variance extracted (AVE) values exceeded 0.50, supporting convergent validity (see Supplementary Table S2). The square roots of AVE for each construct were greater than the corresponding inter-construct correlations, indicating adequate discriminant validity.

To assess potential common method variance associated with self-reported psychosocial measures, a single-factor CFA model was compared with the hypothesized multi-factor model. The single-factor model demonstrated substantially poorer fit, suggesting that common method bias was unlikely to account for the observed associations.

### 3.4 Structural model (SEM) and indirect association

**Figure 4:**
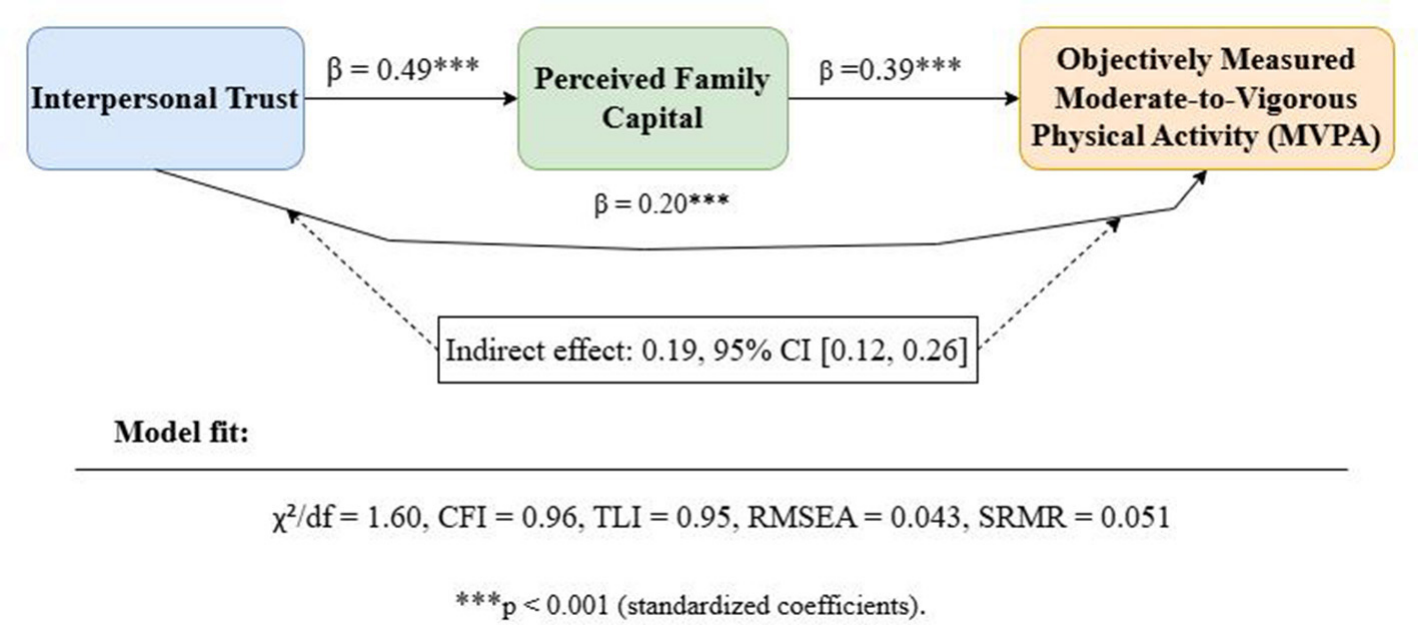
Structural equation model of interpersonal trust, family capital, and MVPA.

The structural equation model demonstrated overall good fit (χ^2^/df = 1.60, CFI = 0.96, TLI = 0.95, RMSEA = 0.043, SRMR = 0.051). Interpersonal trust was positively associated with family capital (β = 0.49, *p* < 0.001). Family capital showed a significant positive statistical association with MVPA (β = 0.39, *p* < 0.001). After adjusting for age, gender, and grade level, interpersonal trust remained significantly associated with MVPA (β = 0.20, *p* < 0.001). Bootstrap results (5,000 resamples) indicated a significant indirect statistical association of interpersonal trust on MVPA through family capital (β = 0.19, 95% CI [0.12, 0.26]). The standardized indirect association represented 48.7% of the total standardized association, as illustrated in Figure 4. In a sensitivity structural equation model additionally adjusting for school, the magnitude and direction of the primary path coefficients remained materially unchanged (Supplementary Table S3).

Given the cross-sectional design of this study, the aforementioned indirect effect should be interpreted solely as a statistical association at the indirect level, not as a causal or temporal relationship. Separate analyses were conducted for weekday and weekend MVPA, and the direction and significance of the primary path coefficients remained consistent across models. Mean MVPA was 49.1 min/day on weekdays and 43.7 min/day on weekends (Supplementary Table S4). Because sedentary time may represent a related behavioral domain rather than a conventional confounder, the sedentary-time-adjusted model was treated as a robustness check. The magnitude and direction of the primary path coefficients remained materially unchanged after additionally adjusting for sedentary time.

### 3.5 Sensitivity analyses

After conducting separate analyses for weekday and weekend MVPA, the direction and significance of the primary path coefficients remained consistent. When sedentary time was further controlled for in the model, the core path results showed no substantial changes, indicating robust model validity.

## 4 Discussion

This study examined the associations among interpersonal trust, family capital, and objectively measured physical activity in adolescents within a social capital theoretical framework. The findings indicated that interpersonal trust was positively associated with MVPA, and family capital was also significantly associated with MVPA. Moreover, family capital accounted for 48.7% of the total association between interpersonal trust and MVPA. These results are consistent with the proposed theoretical model but should be interpreted in light of the cross-sectional design.

The observed association between interpersonal trust and objectively measured MVPA is consistent with social capital theory, which links trust to patterns of social participation (10, 21) Adolescent physical activity frequently occurs within peer interactions or team-based contexts, where interpersonal dynamics may be relevant. Adolescents reporting higher levels of trust may be more likely to engage in socially embedded activity settings, which could be reflected in higher MVPA levels.

Because MVPA was assessed using accelerometer-based objective measurement, the likelihood of shared self-report variance between psychosocial constructs and physical activity outcomes is reduced. At the same time, physical activity experiences may also contribute to social interaction patterns that shape trust perceptions. Therefore, the temporal ordering of these associations cannot be determined within the present cross-sectional design.

Perceived family capital was also positively associated with MVPA (23). As a multidimensional construct encompassing economic, social, and cultural dimensions, perceived family capital reflects adolescents' evaluations of the resources and relational context within the household environment. These dimensions may be statistically associated with physical activity through various contextual mechanisms, including access to facilities, parental involvement, and the transmission of health-related values (22).

The observed indirect statistical association is consistent with the interpretation that psychosocial dispositions and family-level contextual resources may be interconnected within a broader social-ecological framework. Importantly, classical social capital theory often conceptualizes family capital as an antecedent condition influencing psychosocial development, including trust formation. Alternative theoretical orderings—such as family capital statistically preceding interpersonal trust—remain plausible and cannot be excluded within the present cross-sectional design. Therefore, the present findings should be interpreted as evidence of statistical interrelationships rather than directional mechanisms.

From a social ecological perspective, adolescent behavior may be understood as embedded within interactions between individual psychological characteristics and contextual resource structures. The present findings suggest that physical activity is statistically associated not only with individual-level dispositions but also with family-level contextual resources. In practical terms, initiatives aimed at promoting adolescent physical activity may consider relational and family contexts alongside environmental infrastructure.

Methodologically, the integration of accelerometer-based objective measurement with structural equation modeling enhances the transparency of behavioral assessment and analytic procedures. The use of standardized processing protocols and pre-defined quality control criteria contributes to reproducibility and facilitates comparison across studies employing similar wearable approaches.

Several limitations should be acknowledged. The cross-sectional design precludes conclusions regarding temporal ordering (4). The convenience sample drawn from two schools in Sichuan Province limits generalizability. Psychosocial variables were assessed via self-report, which may introduce perceptual bias. Certain contextual factors were not included in the model. In addition, because excluded participants reported lower levels of interpersonal trust and perceived family capital, compliance-related selection bias may have modestly influenced the magnitude of the reported associations. Future research employing longitudinal designs, multi-site sampling frameworks, and multi-informant assessments would help clarify directionality and extend these findings.

Overall, the study provides evidence that interpersonal trust and perceived family capital are statistically associated with adolescents' objectively measured MVPA. The indirect statistical association observed through family capital suggests that psychosocial and family-level contextual resources may be interrelated within a broader relational framework. These findings contribute to understanding patterns linking psychosocial dispositions and contextual resources with adolescent physical activity and highlight the relevance of multilevel social environments in behavioral research.

## 5 Conclusion

This study examined the statistical associations among interpersonal trust, perceived family capital, and adolescents' objectively measured moderate-to-vigorous physical activity (MVPA) within a social capital framework. Adopting a multilevel perspective on social resources, the findings indicate that both interpersonal trust and perceived family-level contextual resources were positively associated with MVPA in the present sample.

The findings indicate that interpersonal trust and perceived family capital were significantly associated with MVPA, with the indirect statistical association through family capital representing a substantial proportion of the total standardized association. This pattern suggests that adolescent physical activity may be statistically related to multiple interrelated psychosocial and contextual factors rather than a single individual-level disposition. These results are consistent with social capital perspectives that emphasize multilevel resource linkages and highlight the relevance of family contexts in adolescent health behavior research.

Methodologically, the integration of accelerometer-based objective measurement with structural equation modeling reduces reliance on self-reported behavioral assessment and enhances transparency in model estimation. The use of standardized processing procedures contributes to reproducibility and facilitates comparison across studies employing similar wearable protocols. The conclusions presented in this study are based strictly on statistical associations and are interpreted with theoretical caution given the constraints of the cross-sectional design.

In summary, this study contributes to the literature by examining statistical associations among interpersonal trust, perceived family capital, and adolescents' objectively measured MVPA within a multilevel social resource framework. The findings highlight the potential interrelationships between psychosocial dispositions and family-level contextual resources in adolescent physical activity.

Future research employing longitudinal designs, multi-site samples, and multi-informant assessments is needed to clarify temporal ordering and to further examine the applicability of social capital perspectives in adolescent health behavior research.

## Data Availability

The datasets presented in this study can be found in online repositories. The names of the repository/repositories and accession number(s) can be found in the article/Supplementary material.
