# Supplementary material for: Interpersonal trust and objectively measured physical activity in adolescents: the mediating role of family capital
Source: Front Public Health. 2026 Apr 24;14:1796300. doi: 10.3389/fpubh.2026.1796300 (PMC13154216; doi:10.3389/fpubh.2026.1796300)
Supplement: Supplementary file 1 [file Supplementary_file_1.docx]

## Supplement

**Supplementary Appendix S1. Accelerometer Data Processing Workflow.**

Accelerometer data were collected using ActiGraph wGT3X-BT devices initialized in ActiLife software (version 6.13.4). The monitors recorded triaxial acceleration at 30 Hz and were worn on the right hip with an elastic belt. Participants were instructed to wear the device from 6:00 a.m. to 11:00 p.m. for seven consecutive days and to remove it only for water-based activities such as bathing or swimming.

After the monitoring period, data were downloaded in ActiLife and prepared for analysis. Raw acceleration signals were converted into activity counts summarized in 60-second epochs. These epoch counts were used for subsequent identification of wear time and activity intensity.

Non-wear periods were identified from the epoch-level count data using the algorithm proposed by Choi et al. Non-wear was defined as at least 90 consecutive minutes of zero counts, allowing short interruptions according to the Choi criteria. A monitoring day was considered valid when wear time reached at least 10 hours. Participants were included in the analysis if they provided at least four valid days of data, including one weekend day.

Activity intensity was classified using the Evenson cut-points developed for children and adolescents. Moderate-to-vigorous physical activity (MVPA) was calculated by summing the minutes spent in moderate and vigorous activity. From the processed data, several variables were derived for analysis, including average daily MVPA, MVPA on weekdays, MVPA on weekends, sedentary time, and indicators describing valid wear time.

**Supplementary Table S1.** Accelerometer data characteristics and quality metrics (N=326).

| **Metric** | **Mean ± SD** | **Range** |
| --- | --- | --- |
| Valid days per participant | 6.2 ± 0.9 | 4-7 |
| Wear time per day (hours) | 14.3 ± 1.8 | 10.0-18.5 |
| Non-wear time per day (hours) | 1.2 ± 0.8 | 0-4.5 |
| Total recorded minutes | 6,048 ± 892 | 4,320-7,200 |
| Valid minutes analyzed | 5,328 ± 756 | 3,600-6,480 |
| Data retention rate (%) | 88.1 ± 6.4 | 75.0-95.2 |
| Average counts per minute | 412 ± 156 | 98-892 |
| Steps per day | 8,247 ± 2,834 | 2,145-16,892 |
| MVPA minutes per day | 47.3 ± 22.1 | 8.5-128.4 |
| Sedentary time per day (hours) | 8.9 ± 1.6 | 4.2-13.1 |

**Supplementary Table S2.** Construct Reliability and Validity.

| **Construct** | **CR** | **AVE** | **√AVE** | **Correlation (Trust–FC)** |
| --- | --- | --- | --- | --- |
| Interpersonal Trust | 0.938 | 0.427 | 0.653 | 0.370 |
| Perceived Family Capital | 0.923 | 0.453 | 0.673 | 0.370 |

**Supplementary Table S3.** Sensitivity structural equation model additionally adjusting for school (N = 326).

| **Path** | **β** | **SE** | **p** |
| --- | --- | --- | --- |
| Interpersonal Trust → Family Capital | 0.37 | 0.05 | <0.001 |
| Family Capital → MVPA | 0.21 | 0.06 | 0.003 |
| Interpersonal Trust → MVPA | 0.14 | 0.07 | 0.034 |
| Indirect effect | 0.08 | 0.03 | 0.009 |

**Note:** This table presents a sensitivity structural equation model additionally adjusting for school (two-school indicator). The magnitude and direction of the primary path coefficients remained materially unchanged compared with the main model.

**Supplementary Table S4.** Weekday and weekend MVPA descriptive statistics (N = 326)

| **Variable** | **Mean ± SD** |
| --- | --- |
| Weekday MVPA (min/day) | 49.1 ± 23.4 |
| Weekend MVPA (min/day) | 43.7 ± 21.6 |

**Note:** MVPA = moderate-to-vigorous physical activity. Values represent mean minutes of MVPA per day.
